# Role of Exogenous Pyruvate in Maintaining Adenosine Triphosphate Production under High-Glucose Conditions through PARP-Dependent Glycolysis and PARP-Independent Tricarboxylic Acid Cycle

**DOI:** 10.3390/ijms252011089

**Published:** 2024-10-15

**Authors:** Hideji Yako, Naoko Niimi, Shizuka Takaku, Ayako Kato, Koichi Kato, Kazunori Sango

**Affiliations:** 1 Diabetic Neuropathy Project, Tokyo Metropolitan Institute of Medical Science, Tokyo 156-8506, Japan; niimi-nk@igakuken.or.jp (N.N.); takaku-sz@igakuken.or.jp (S.T.); 2 Laboratory of Medicine, Aichi Gakuin University School of Pharmacy, Nagoya 464-8650, Japan; k-ayako@dpc.agu.ac.jp (A.K.); kkato@dpc.agu.ac.jp (K.K.)

**Keywords:** exogenous pyruvate, high-glucose, Schwann cells, cell death, PARP, glycolysis, tricarboxylic acid cycle, adenosine triphosphate depletion

## Abstract

Pyruvate serves as a key metabolite in energy production and as an anti-oxidant. In our previous study, exogenous pyruvate starvation under high-glucose conditions induced IMS32 Schwann cell death because of the reduced glycolysis–tricarboxylic acid (TCA) cycle flux and adenosine triphosphate (ATP) production. Thus, this study focused on poly-(ADP-ribose) polymerase (PARP) to investigate the detailed molecular mechanism of cell death. Rucaparib, a PARP inhibitor, protected Schwann cells against cell death and decreased glycolysis but not against an impaired TCA cycle under high-glucose conditions in the absence of pyruvate. Under such conditions, reduced pyruvate dehydrogenase (PDH) activity and glycolytic and mitochondrial ATP production were observed but not oxidative phosphorylation or the electric transfer chain. In addition, rucaparib supplementation restored glycolytic ATP production but not PDH activity and mitochondrial ATP production. No differences in the increased activity of caspase 3/7 and the localization of apoptosis-inducing factor were found among the experimental conditions. These results indicate that Schwann cells undergo necrosis rather than apoptosis or parthanatos under the aforementioned conditions. Exogenous pyruvate plays a pivotal role in maintaining the flux in PARP-dependent glycolysis and the PARP-independent TCA cycle in Schwann cells under high-glucose conditions.

## 1. Introduction

Diabetic peripheral neuropathy (DPN) is the earliest and most frequent chronic complication of diabetes mellitus, affecting approximately 36% of Japanese patients with type 2 diabetes [1]. The pathogenesis of DPN remains elusive, and no effective disease-modifying remedies have been developed to date. Prolonged hyperglycemia enhances glucose uptake in Schwann cells and dorsal root ganglion (DRG) neurons, where excessive glucose changes the flux in glycolysis, collateral glycolytic pathways (i.e., polyol, hexosamine, diacylglycerol, and advanced glycation end-product pathways), and the tricarboxylic acid (TCA) cycle. Apart from glucose metabolism, lipid metabolism is also altered under these conditions. These metabolic alterations elicit oxidative stress, endoplasmic reticulum stress, inflammation, and mitochondrial damage [2,3,4,5,6]. The activation of poly-(ADP-ribose) polymerase (PARP) 1 also plays a role in the development and progression of DPN, and the inhibition of PARP1 is a potential therapeutic strategy against DPN [7,8,9,10,11,12,13,14].

Endogenous pyruvate is a key metabolite in adenosine triphosphate (ATP) production, which links glycolysis and the TCA cycle, whereas exogenous pyruvate serves as an anti-oxidant and anti-inflammatory molecule [15,16]. Pyruvate supplementation has been shown to ameliorate the retinopathy and nephropathy of diabetic animals through its anti-oxidant and anti-inflammatory activities [17,18,19,20]. However, no studies have explored the efficacy of pyruvate toward DPN. In our previous reports, pyruvate starvation under high-glucose (>10 mM) conditions induced the rapid cell death of immortalized mouse Schwann cells (IMS32) and primary cultured adult rat DRG neurons, which indicates the pivotal role of pyruvate in maintaining glycolysis–TCA cycle flux under hyperglycemia [21]. These findings demonstrate that exogenous pyruvate has beneficial effects on the metabolism and survival in extensive cell types such as immortalized and primary cultured cells. Rucaparib, a PARP inhibitor, also restored the cell viability and glycolytic flux through the recovery of glyceraldehyde-3-phosphate dehydrogenase (GAPDH) activity under high-glucose pyruvate-starved conditions [21]. However, whether exogenous pyruvate maintains glycolysis–TCA cycle flux and the mechanism by which Schwann cells undergo cell death by pyruvate starvation under high-glucose conditions remain unclear. In the present study, using IMS32 Schwann cells, pyruvate starvation under high-glucose conditions inhibited the glycolytic flux in a PARP-dependent manner, as well as the pyruvate dehydrogenase (PDH) activity and TCA cycle flux in a PARP- and pyruvate dehydrogenase kinase (PDK)-independent manner, respectively. These metabolic changes resulted in ATP depletion and necrosis-like cell death.

## 2. Results

### 2.1. Pyruvate Starvation under High-Glucose Conditions Decreased Mitochondrial ATP Production by Impaired PDH Activity in PDK- and PARP-Independent Manners

**Figure 1:**
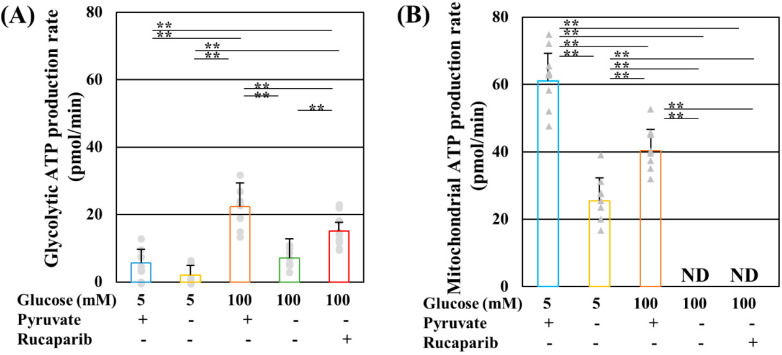
Rucaparib ameliorated glycolytic, but not mitochondrial, adenosine triphosphate (ATP) production under high-glucose and pyruvate-starved conditions. Glycolytic (**A**) and mitochondrial (**B**) ATP production of IMS32 cells under exposure to 5 mM glucose in the presence (**blue**) and absence (**yellow**) of pyruvate conditions, 100 mM glucose in the presence (**brown**) and absence (**green**) of pyruvate conditions, and 100 mM glucose in the absence of pyruvate conditions supplemented with rucaparib (**red**) were determined. Values represent mean + SD from nine to ten experiments (individual values in glycolytic and mitochondrial ATP production are depicted as circles and triangles, respectively). ** *p* < 0.01. ND: not detected.

In our previous study, rucaparib ameliorated cell viability, intracellular ATP contents, and glycolytic flux, but not mitochondrial respiration, in IMS32 Schwan cells under high-glucose pyruvate-starved conditions [21]. Consistent with the aforementioned findings, glycolytic and mitochondrial ATP production assessed by the Extracellular Flux Analyzer and Real-Time ATP rate assay was reduced by pyruvate starvation under normal and high-glucose conditions, and rucaparib remarkably recovered glycolytic, but not mitochondrial, ATP production under high-glucose pyruvate-starved conditions (Figure 1). The levels of total ATP production were nearly comparable between normal- and high-glucose conditions in the presence of pyruvate (Figure 1).

**Figure 2:**
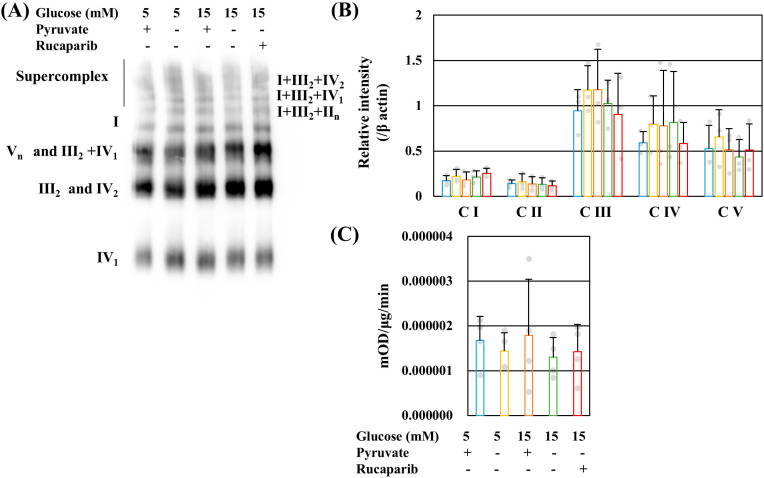
Pyruvate starvation and rucaparib had no significant effects on oxidative phosphorylation under high-glucose conditions. Representative image of blue native PAGE and Western blotting of the supercomplex of IMS32 cells under exposure to 5 mM glucose in the presence and absence of pyruvate, 15 mM glucose in the presence and absence of pyruvate, and 15 mM glucose in the absence of pyruvate conditions supplemented with rucaparib (**A**). The bands of the complex in the electric transporter chain were estimated as referred to in the manuscript [26]. Relative complex I (CI; NDUFB8), CII (SDHB), CIII (MTCO1), CIV (UQCR2), and CV (ATP5A) expression (**B**) and CI activities (**C**) were measured. The representative blots for these complexes and β actin (**B**) are shown in Figure S1. (**B**,**C**) These bars exhibit the conditions of 5 mM glucose in the presence (**blue**) and absence (**yellow**) of pyruvate conditions, 15 mM glucose in the presence (**brown**) and absence (**green**) of pyruvate conditions, and 15 mM glucose in the absence of pyruvate conditions supplemented with rucaparib (**red**). The values represent the mean + SD from three (**B**) and four (**C**) experiments (individual values are depicted as circles).

In elucidating the mechanisms of impaired mitochondrial ATP production under high-glucose pyruvate-starved conditions, the supercomplex (SC) of the electric transporter chain (ETC) was assessed. Complexes I–V (CI–CV) are constituents of ETC, and SC is formed by CI, CIII, and CIV. SC could reduce ROS [22,23] and increase transfer efficiency [24,25]. Blue native PAGE and Western blot analyses revealed that pyruvate starvation and rucaparib supplementation did not change the SC profile under normal and high-glucose conditions (Figure 2A). No significant differences in the amount of CI–CV (Figure 2B) or the activity of CI (Figure 2C) were found among these conditions. These ETC and complex data demonstrate that oxidative phosphorylation (OXPHOS) functions properly under high-glucose pyruvate-starved conditions.

**Figure 3:**
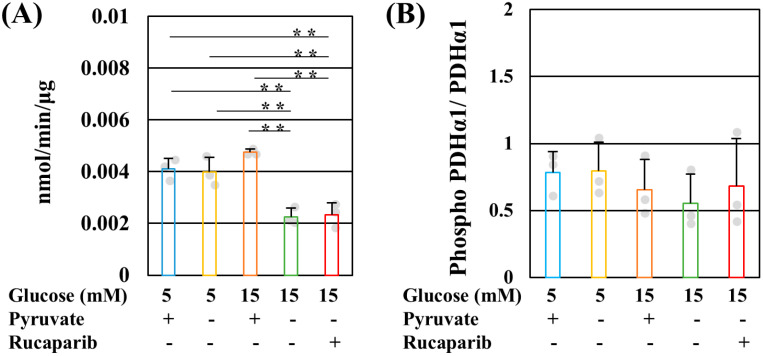
Pyruvate starvation decreased pyruvate dehydrogenase (PDH) activity without PDHE1α phosphorylation under high-glucose conditions. PDH activity (**A**) and PDHE1α (Ser293) phosphorylation (**B**) of IMS32 cells under exposure to 5 mM glucose in the presence (**blue**) and absence (**yellow**) of pyruvate conditions, 15 mM glucose in the presence (**brown**) and absence (**green**) of pyruvate conditions, and 15 mM glucose in the absence of pyruvate conditions containing rucaparib (**red**) were determined. The representative blots for PDHα1 (Ser293) and PDH (**B**) are shown in Figure S2. The values represent the mean + SD from three experiments (individual values are depicted as circles). ** *p* < 0.01.

PDH catalyzes the conversion of pyruvate to acetyl CoA [27]. Reduced PDH activity under high-glucose pyruvate-starved conditions was not ameliorated by rucaparib (Figure 3A). PDK mediates PDH phosphorylation, which reduces the enzymatic activity [27]. PDH E1α (Ser293) phosphorylation is also increased in diabetic DRG [28]. In contrast to the decreases in PDH activity, the phosphorylation level of PDH E1α (Ser293) remained unchanged under these conditions (Figure 3B). We further confirmed PDH regulation using dichloroacetate (DCA), a PDK inhibitor. DCA failed to rescue IMS32 cell death under high-glucose pyruvate-starved conditions even in the presence of rucaparib (Figure S3). These results indicate that PDK did not regulate PDH activity and the decrease in PDH activity impairs mitochondrial ATP production under high-glucose pyruvate-starved conditions.

### 2.2. GAPDH Did Not Receive Poly-ADP-ribosylation under High-Glucose Pyruvate-Starved Conditions

**Figure 4:**
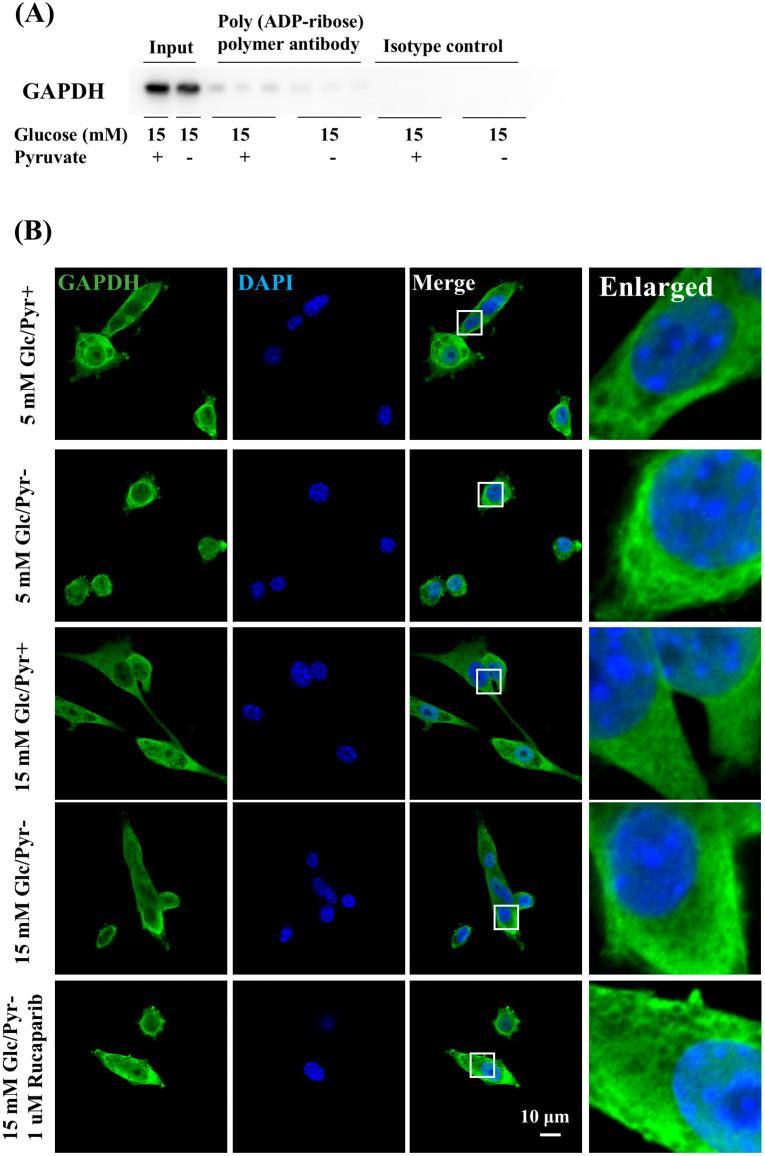
GAPDH was not poly-ADP-ribosylated under high-glucose pyruvate-starved conditions. (**A**) The degrees of poly-ADP-ribosylation of glyceraldehyde-3-phosphate dehydrogenase (GAPDH) under high-glucose conditions in the presence and absence of the pyruvate condition were assessed by the immunoprecipitation of poly-(ADP-ribose) polymer or isotype control and Western blotting of GAPDH. Blots for GAPDH (**A**) and the image of the Ponceau stain are shown in Figure S4. (**B**) Images of the immunostaining of GAPDH (**green**) and nuclear staining of DAPI (**blue**) of IMS32 cells under 5 or 15 mM glucose in the presence and absence of pyruvate and 15 mM glucose in the absence of pyruvate containing rucaparib. Enlarged images of the white box in merged images are shown. Scale bar: 10 μm.

In our previous studies, a decrease in GAPDH activity under high-glucose conditions in the absence of pyruvate was recovered by rucaparib treatment [21]. Moreover, GAPDH has been reported to receive poly-ADP-ribosylation mediated by PARP1 and to decrease the activity [29]. Therefore, whether pyruvate starvation under high-glucose conditions induced the poly-ADP-ribosylation of GAPDH was examined in this study using immunoprecipitation. Figure 4A shows the immunoprecipitation of poly-(ADP-ribose) (PAR) and the visualization of GAPDH, indicating that no differences in poly-ADP-ribosylated GAPDH were found between the presence and absence of pyruvate under hyperglycemia. Thus, the translocation of GAPDH to nuclei is necessary to receive poly-ADP-ribosylation mediated by PARP1 because PARP1 is a nuclear protein [30]. Whether GAPDH was present in nuclei was also assessed by using immunocytochemistry. GAPDH signals were detected in the cytoplasm, but not nuclei, under normal- and high-glucose conditions in the presence and absence of pyruvate supplemented with rucaparib (Figure 4B). Our previous [21] and present results indicate that the impaired GAPDH activity does not result from its poly-ADP-ribosylation mediated by PARP1.

### 2.3. Pyruvate-Starvation-Induced Necrosis-like Schwann Cell Death under High-Glucose Conditions

**Figure 5:**
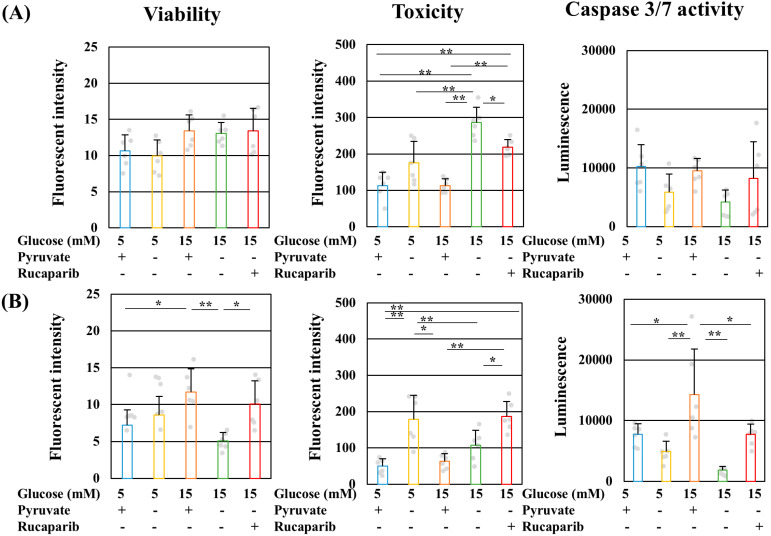

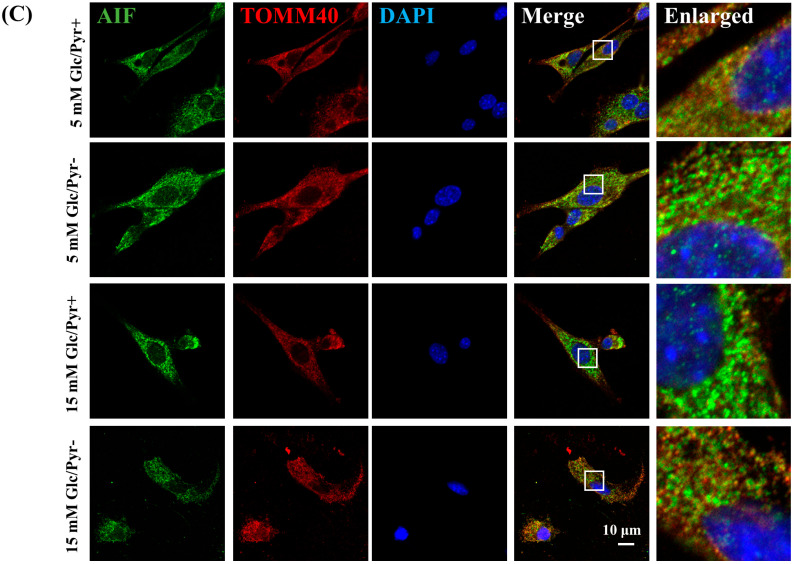
Pyruvate-starvation-induced necrosis-like cell death under high-glucose conditions. The cell viability, toxicity, and caspase 3/7 activity of IMS32 cells at 3 (**A**) and 6 (**B**) h under exposure to 5 mM glucose in the presence (**blue**) and absence (**yellow**) of pyruvate conditions, 15 mM glucose in the presence (**brown**) and absence (**green**) of pyruvate conditions, and 15 mM glucose in the absence of pyruvate conditions supplemented with rucaparib (**red**) were determined. (**C**) Images of immunostaining for apoptosis-inducing factor (AIF; **green**) and TOMM40 (**red**) and nuclear staining of DAPI (**blue**) of IMS32 cells under 5 or 15 mM glucose in the presence and absence of pyruvate. Enlarged images of the white box in merged images are shown. Scale bar: 10 μm. The values represent the mean + SD from six experiments (individual values are depicted as circles). * *p* < 0.05, ** *p* < 0.01.

PARP1-associated cell death is classified into three patterns, namely, apoptosis-inducing factor (AIF)-induced cell death (parthanatos), caspase3-activated cell death (apoptosis), and energy-depletion-induced cell death (necrosis) [31]. The cell viability, cell toxicity, caspase3/7 activity (Figure 5A,B), and intracellular AIF localization (Figure 5C) were assessed to elucidate the mechanism of high-glucose pyruvate-starved IMS32 cell death. Pyruvate starvation under high-glucose conditions increased cell toxicity at 3 h (Figure 5A) and then decreased cell viability at 6 h (Figure 5B). In both exposure time periods, no significant increases in caspase3/7 activities were observed under these conditions (Figure 5A,B). Rucaparib treatment under high-glucose pyruvate-starved conditions ameliorated cell viability, reduced toxicity, and prevented the decrease in caspase3/7 activity (Figure 5A,B). Parthanatos is induced by the accumulation of PAR and the translocalization of AIF from the mitochondria to nuclei [32]. In assessing the possibility of parthanatos, immunocytochemistry was conducted using antibodies to AIF and a mitochondrial marker, namely, TOMM40. AIF signals were observed in IMS32 cells exposed to normal glucose conditions in the presence of pyruvate (Figure 5C, upper panels). Most of these signals colocalized with TOMM40 signals, indicating that AIF existed in the mitochondria under normal- and high-glucose conditions in the presence and absence of pyruvate (Figure 5C). AIF signals under high-glucose pyruvate-starved conditions were detected in the cytoplasm but not in nuclei (Figure 5C, lower panels). In examining the possibility of pyruvate-starvation-induced parthanatos, PAR profiles were assessed using immunocytochemistry and Western blotting. PAR signals were not accumulated in nuclei, and no significant differences in the amount of PAR were found among these conditions (Figures S5 and S6). These results indicate the occurrence of necrosis-like cell death under high-glucose pyruvate-starved conditions.

## 3. Discussion

Pyruvate is an essential metabolite for ATP production under aerobic and anaerobic conditions. We have reported that exogenous pyruvate starvation under high-glucose (>10 mM) conditions induced rapid IMS32 Schwann cell death [21]. In this study, the precise mechanism of energy depletion leading to necrosis-like cell death under such conditions was further demonstrated.

PARP1 is a nuclear protein [30]; thus, GAPDH translocation into nuclei is prerequisite for its poly-ADP-ribosylation mediated by PARP1. By contrast, nuclear GAPDH activates PARP1 [33]. In our study, neither increases in nuclear GAPDH and PAR nor the poly-ADP-ribosylation of GAPDH was detected under high-glucose pyruvate-starved conditions. These findings do not support the idea that PARP1 directly modulates GAPDH activity under those conditions, although rucaparib ameliorated GAPDH activity. Rucaparib has been shown to suppress not only PARP1 but also other PARPs, including PARP2, PARP3, PARP4, PARP10, PARP15, PARP16, TNKS1, and TNKS2 [34]. It was hypothesized that these PARPs may exert a direct or indirect influence on GAPDH activity under these conditions. However, it is challenging to identify the specific PARPs and the underlying mechanisms because of the vast number of potential candidates and no differences in PAR profile. The PARP10-mediated mono-ADP-ribosylation of GAPDH has no impact on its activity [35], although whether other PARPs mediate GAPDH activity remains unclear. Therefore, GAPDH activity may directly or indirectly be modulated by PARPs under high-glucose pyruvate-starved conditions.

ATP is downregulated during necrosis [36,37] and parthanatos [38] but upregulated during apoptosis [39]. Previous [21] and present studies revealed that high-glucose pyruvate-starved conditions reduced the levels of lactate, ATP, NAD, NADH, and ECAR and the caspase3/7 activity, without affecting the AIF localization and PAR profiles. Moreover, rucaparib recovered ATP, NAD, NADH, and ECAR. In the Extracellular Flux Analyzer, ECAR (i.e., glycolysis) measures H+ originating from lactate in cultured media. These results suggested that PARP inhibition improved lactate production via glycolysis under these conditions. These results indicate that Schwann cells undergo necrotic cell death under those conditions, and rucaparib may prevent energy depletion by ameliorating glycolytic ATP production.

Compared with glycolytic ATP production, rucaparib treatment failed to restore mitochondrial ATP production. N-methyl-N-nitroso-N-nitroguanidine-induced PARP1 activation in the absence of the pyruvate condition reduced glycolysis and mitochondrial respiration by inhibiting hexokinase rather than depleting NAD [38]. Moreover, pyruvate supplementation recovered the PARP1-mediated reduction in mitochondrial respiration [38]. Our experimental condition also reduced hexokinase activity, whereas rucaparib treatment recovered NAD and NADH levels [21]. Therefore, pyruvate is an important metabolite in glycolytic and mitochondrial ATP production, and there are more pathogeneses in addition to the PARP-mediated pathway in mitochondrial dysmetabolism under high-glucose pyruvate-starved conditions. A decrease in PDH activity is a pathogenesis of mitochondrial failure. PDH activity is modulated by PDK-mediated phosphorylation. However, PDH E1α phosphorylation in Ser293, which is a main phosphorylation site mediated by PDK, did not show remarkable changes under our experimental conditions. Moreover, DCA, a PDK inhibitor, also failed to rescue the cell death induced by pyruvate starvation under hyperglycemia. The *O*-glycosylation of E2 and E3 subunits in the PDH complex also modulates PDH activity in the heart [40]. *O*-linked N-acetylglucosamine, which is a substrate in *O*-glycosylation, is produced in collateral glycolytic pathways such as the hexosamine pathway [41]. The hexosamine pathway is a pathogenesis of diabetic neuropathy [41], and it enhances the flux under hyperglycemia accompanied with a decrease in GAPDH activity [29,42]. Under high-glucose conditions in the absence of pyruvate, collateral glycolytic pathways, including the hexosamine pathway, could be accelerated because of decreases in GAPDH activity [21]. Therefore, PDH activity may be reduced by *O*-glycosylation. However, examining whether E2 and E3 subunits in the PDH complex receive *O*-glycosylation and other post-translational modifications such as succination is necessary [43].

SC in ETC plays an important role in ATP production during OXPHOS, accompanied with ROS production. However, SC shows discrepant findings under diabetic conditions. SC in the muscle of patients with type 2 diabetes was reduced [44], whereas SC in the liver of streptozotocin-induced diabetic rats was not changed [45]. Short-term hyperglycemia (1 h) in Schwann cells did not affect SC and CI–CV in the presence and absence of pyruvate. However, whether long-term hyperglycemia affects SC in Schwan cells remains unclear. Therefore, further study is necessary to investigate the impact of SC on the pathogenesis of DPN.

Although pyruvate activated the TCA cycle via inhibiting PDK [46], DCA failed to rescue IMS32 cell death under high-glucose pyruvate-starved conditions, indicating that enzyme activity other than PDH may be inhibited. In addition to decreased PDH activity, hexokinase and GAPDH activities were also reduced under these conditions, resulting in decreased glycolytic flux [21]. OCRs under these conditions were nearly undetectable, even though rucaparib supplementation improved ECAR [21]. These data suggested the impaired enzyme activity and flux in the TCA cycle. Since OXPHOS was not affected by high-glucose pyruvate-starved conditions, there is a possibility of a reduced enzyme activity in the TCA cycle. A candidate for the reduced enzyme activity is aconitase, an enzyme catalyzing the conversion of citrate to isocitrate in the TCA cycle. It was reported that the aconitase activity was inhibited by ROS in diabetic sciatic nerves [47]. Elevating ROS production under pyruvate starvation under hyperglycemia was also observed [21]. Therefore, it is plausible that the impaired glycolytic flux was due to the decreased enzyme activities such as hexokinase, GAPDH resulted in the reduction in pyruvate levels, and the impaired TCA cycle flux due to the decreased enzyme activity such as aconitase resulted in undetectable mitochondrial ATP production, even though the PDH activity remained at 50%.

Evidence shows that the serum pyruvate concentration increases under diabetes conditions [48,49,50]; however, recent reports show decreased pyruvate concentration in serum among streptozotocin-induced diabetic rats [51,52,53,54] and patients with type 2 diabetes [55], compared with those without diabetes. In addition, the serum pyruvate concentration is lower in patients with diabetic complications than in those without complications [55]. The mechanism of regulating pyruvate levels in serum under diabetes conditions remains unclear, but diabetes and its complications may decrease serum pyruvate concentration. In vivo studies of the efficacy of pyruvate on DPN are currently in progress in our laboratory and will be submitted in the near-future. Further study is needed to investigate the regulation of pyruvate metabolism in diabetes and its complications.

**Figure 6:**
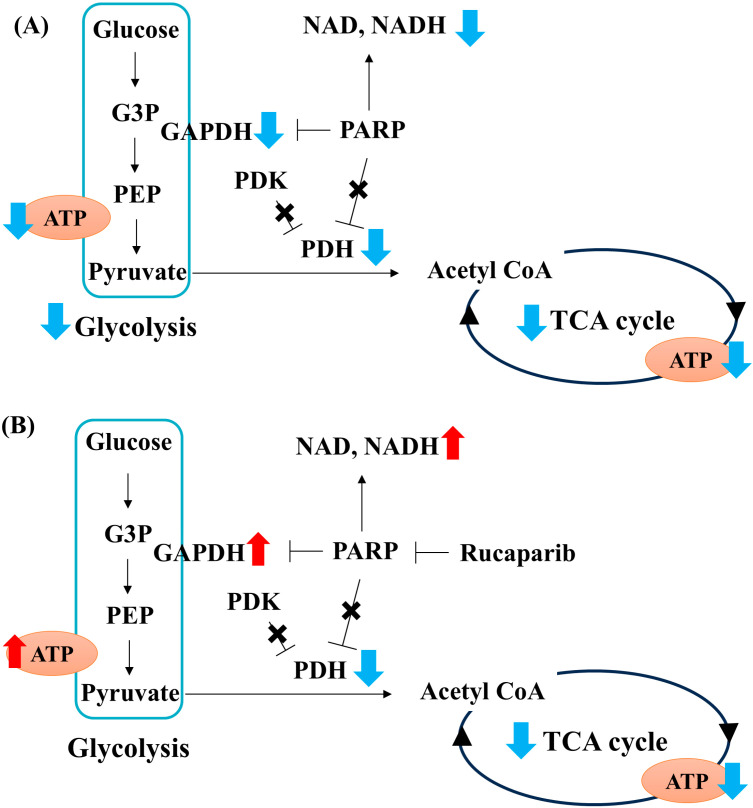
The schematic representation of metabolic changes in IMS32 cells under high-glucose pyruvate-starved conditions. Pyruvate starvation under high-glucose conditions resulted in impaired glycolysis–tricarboxylic acid (TCA) cycle flux and adenosine triphosphate (ATP) production due to the impaired glyceraldehyde-3-phosphate dehydrogenase (GAPDH) and pyruvate dehydrogenase (PDH) activities (**A**). Rucaparib treatment under these conditions led to the recovery of NAD, NADH, GAPDH activity, and glycolytic flux and ATP production. However, PDH activity, TCA cycle flux, and mitochondrial ATP production remained unaltered (**B**). PDH activity was found to be independent of PARP and PDH kinase (PDK). These metabolic changes resulted in necrosis-like IMS32 cell death. Blue allows: decrease, Red allows: increase, ×: no regulation, G3P: glyceraldehyde-3-phosphate, PEP: phosphor enol pyruvate.

The findings in this study indicate that pyruvate starvation under high-glucose conditions decreased the flux in glycolysis and the TCA cycle through the PARP-GAPDH pathway and PDH activity, respectively. These metabolic alterations led to the failure of mitochondrial ATP production in PDK- and PARP-independent manners. Then, ATP depletion induced necrotic Schwann cell death. Based on present and previous [21] studies, exogenous pyruvate plays a pivotal role in maintaining glycolysis–TCA cycle flux and ATP production by regulating PARP-dependent GAPDH activity (glycolysis) as well as PDK- and PARP-independent PDH activity (TCA cycle) under high-glucose conditions (Figure 6).

## 4. Materials and Methods

### 4.1. Cell Culture

In this study, spontaneously immortalized adult mouse IMS32 Schwann cells have been established and passaged by our laboratory (formerly known as the ALS/neuropathy project). Dulbecco’s modified Eagle’s medium (DMEM) containing 5.6 mM glucose and 1 mM sodium pyruvate (Nacalai Tesque Inc., Kyoto, Japan) supplemented with 5% fetal bovine serum (FBS; Thermo Fisher Scientific Inc., Waltham, MA, USA) and antibiotic–antimycotic mixed solution (100 unit/mL of penicillin, 100 μg/mL of streptomycin, and 250 ng/mL of amphotericin B; Nakarai Tesque Inc., Kyoto, Japan) was used for the maintenance and passage of IMS32 cells. Glucose- and pyruvate-free DMEM (Thermo Fisher Scientific, Waltham, MA, USA. #11966025) supplemented with 5% FBS and antibiotic–antimycotic mixed solution was also used as a basal medium in various assays.

### 4.2. Measurement of Glycolytic and Mitochondrial ATP Production

Glycolytic and mitochondrial ATP production was assessed using the xFe96 Extracellular Flux Analyzer (Agilent Technologies, Santa Clara, CA, USA) and Real-Time ATP Rate Assay kit (Agilent Technologies, Santa Clara, CA, USA) in accordance with the manufacturer’s manual. In brief, IMS32 cells were seeded on a 96-well plate at a density of 1 × 10^4^ cells and allowed to adhere overnight. The cells were incubated for 1 h at 37 °C in DMEM (Agilent Technologies, Santa Clara, CA, USA) containing 5 or 100 mM glucose (Sigma–Aldrich Co. LCC, St Louis, MO, USA) in the presence or absence of 1 mM sodium pyruvate (Sigma, LCC, St Louis, MO, USA) supplemented with 1 μM rucaparib (Selleck Chemicals, Houston, TX, USA). It became evident that the high cell density (1 × 10^4^ cells/well) was required for the measurement of OCR and ECAR in the xFe96 Extracellular Flux Analyzer, whereby higher glucose is necessary to induce cell death in a higher cell density. The Seahorse assays were conducted under 100 mM glucose conditions, in a similar manner to our previous study [21]. Glycolytic and mitochondrial ATP production was evaluated by sequential oligomycin and rotenone/antimycin A injection. The final concentrations of oligomycin and rotenone/antimycin A used in the experiment were 1.5 μM and 0.5 μM, respectively.

### 4.3. Measurement of Cell Viability, Toxicity, and Caspase 3/7 Activity

IMS32 cells were seeded on a 96-well white plate at a density of 3 × 10^3^ cells and maintained for 3 or 6 h under 5 or 15 mM glucose in the presence or absence of 1 mM pyruvate containing 1 μM rucaparib. The viability, toxicity, and caspase 3/7 activity of IMS32 cells under each culture condition were assessed using the ApoTox-GloTM Triplex Assay in accordance with the manufacturer’s instructions (Promega, Madison, WI, USA).

### 4.4. MTS Assay

IMS32 cells were seeded on a 96-well white plate at a density of 3 × 10^3^ cells and maintained for 24 h under 5 or 15 mM glucose in the presence or absence of 1 mM pyruvate containing 1 μM rucaparib and 1, 5, and 10 mM dichloroacetate (Sigma–Aldrich Co. LCC, St Louis, MO, USA). The cell viability of IMS32 cells under each culture condition was assessed using the CellTiter 96 AQueous One Solution Cell Proliferation Assay kit in accordance with the manufacturer’s instructions (Promega, Madison, WI, USA).

### 4.5. Western Blotting

Western blot analysis was performed as previously described [21]. In brief, IMS32 cells were seeded on 15 cm dishes at a density of 1.1 × 10^6^ cells and maintained for 1 h under 5 or 15 mM glucose in the presence or absence of 1 mM pyruvate containing 1 μM rucaparib. The cells were dissolved in RIPA buffer (FUJIFILM Wako Pure Chemical Corp., Osaka, Japan) containing a protease inhibitor cocktail (Takara Bio Inc., Shiga, Japan) and sonicated using a handy sonicator (TOMY SEIKO Co., Ltd., Tokyo, Japan). Protein electrophoresis and transfer were performed by the NuPAGE system (Thermo Fisher, Waltham, MA, USA). The efficiency of protein transfer was assessed by Ponceaus S staining (Cell Signaling Technology, Danvers, MA, USA). Target proteins were visualized using the following antibodies: rabbit anti-GAPDH polyclonal antibody (1:5000; Sigma–Aldrich Co. LCC, St Louis, MO, USA); mouse anti-poly-(ADP-ribose) polymer monoclonal antibody (1:1000; Abcam, Cambridge, UK); Total OXPHOS Rodent WB Antibody Cocktail (1:1000; Abcam, Cambridge, UK); rabbit anti-Phospho PDHα1 (Ser293) monoclonal antibody (1:1000; Cell Signaling Technology); rabbit anti-PDH monoclonal antibody (1:1000; Cell Signaling Technology); mouse anti-β-actin monoclonal antibody (1:4000; Sigma–Aldrich Co. LCC, St Louis, MO, USA); and horseradish-peroxidase-conjugated anti-mouse IgG and anti-rabbit IgG (1:4000, MBL Corp., Ltd., Nagoya, Japan). The bands of interest were visualized using the ECL plus Western blotting detection kit (GE Healthcare, Chicago, IL, USA) and ImmunoStar LD (FUJIFILM Wako Pure Chemical Corp., Richmond, VA, USA). The signal intensity was quantified using the chemiluminescence imaging system EZ capture II (ATTO, Tokyo, Japan) and ImageJ software, version 1.54f, and the relative intensity of each protein is expressed as the intensity of each protein divided by the intensity of β-actin or PDH.

### 4.6. Immunoprecipitation

IMS32 cells were seeded on 15 cm dishes at a density of 1.1 × 10^6^ cells and maintained for 1 h under 15 mM glucose in the presence or absence of 1 mM pyruvate. The cell lysate was prepared as described in Western blotting. After washing the magnetic beads (Dynabeads protein G, Thermofisher, Waltham, MA, USA), 3 μg of poly-(ADP-ribose) polymer antibody (Abcam, Cambridge, UK) or isotype control (Abcam, Cambridge, UK) was added to the beads. The beads and antibodies were incubated at room temperature with rotation for 10 min. After washing with PBST, 25 μg of lysate was added in the bead–antibody complex and incubated at room temperature with rotation for 60 min. The beads were washed with PBST and eluted in LDL sample buffer (Thermofisher, Waltham, MA, USA) at 70 °C for 10 min. The immunoprecipitated proteins were further processed as described in Western blotting.

### 4.7. Immunocytochemistry

IMS32 cells were seeded at a density of 3 × 10^3^ cells and maintained for 1 h under 5 or 15 mM glucose in the presence or absence of 1 mM pyruvate containing 1 μM rucaparib. After washing with PBS, the cells were fixed with 4% PFA (Nacalai Tesque Inc., Kyoto, Japan) for 10 min. Then, the cells were immersed with 0.4% Block Ace (DS Pharma Biomedical, Osaka, Japan) containing 0.1% Triton X-100 for 10 min because of permeabilization and blocking. Target proteins were visualized using the following antibodies: rabbit anti-AIF polyclonal antibody (1:500; Proteintech Group, Rosemont, IL, USA), mouse anti-TOMM40 monoclonal antibody (1:1000; Proteintech Group, Rosemont, IL, USA), rabbit anti-GAPDH polyclonal antibody (1:5000; Sigma–Aldrich Co. LCC, St Louis, MO, USA), mouse anti-poly-(ADP-ribose) polymer monoclonal (1:400; Abcam, Cambridge, UK), and chicken Alexa 488 or 594 conjugated-anti-mouse and rabbit IgG antibodies (1:200; Thermo Fisher, Waltham, MA, USA). The cells were enclosed with DAPI Fluoromount-G (SouthernBiotech, Birmingham, AL, USA). Fluorescent images were observed under a confocal laser scan microscope (LSM780, Carl Zeiss, Jena, Germany).

### 4.8. Mitochondrial Isolation and Blue Native PAGE

Mitochondrial isolation and blue native PAGE were referred to in [26]. In brief, IMS32 cells were seeded on 15 cm dishes at a density of 1.1 × 10^6^ cells and maintained for 1 h under 5 or 15 mM glucose in the presence and absence of 1 mM pyruvate containing 1 μM rucaparib. The cells collected from 3 or 4 dishes were resuspended in 10 mM Tris/1 mM EDTA buffer containing 200 mM sucrose (isolation buffer). Then, the cells were homogenized with a glass homogenizer and centrifuged at 600× *g* and 4 °C for 10 min. Supernatants were centrifuged at 7000× *g* and 4 °C for 10 min. Pellets were resolved in the isolation buffer, followed by centrifugation at 7000× *g* and 4 °C for 10 min. Pellets were resuspended in the sample buffer (Thermofisher, Waltham, MA, USA) containing 0.5% Digitonin (Thermofisher, Waltham, MA, USA). After incubating on ice for 20 min, lysates were centrifuged at 20,000× *g* and 4 °C for 10 min. Coomassie Brilliant Blue (CBB) G-250 was added in supernatants. Proteins were separated on native PAGE 3–12% gradient gel (Thermofisher, Waltham, MA, USA) with native PAGE running buffer containing CBB G-250. After electrophoresis, the gels were further processed for the protein transfer and visualization of SC as described in Western blotting.

### 4.9. Measurement of PDH and Complex I Activity

IMS32 cells were seeded on 15 cm dishes at a density of 1.1 × 10^6^ cells and maintained for 1 h under 15 mM glucose in the presence or absence of 1 mM pyruvate containing 1 μM rucaparib. PDH and complex I activities were assessed using the Pyruvate Dehydrogenase Activity Assay Kit (Sigma–Aldrich Co. LCC, St Louis, MO, USA) and Complex I Enzyme Activity Assay Kit (Abcam, Cambridge, UK), respectively, in accordance with the manufacturer’s instructions.

### 4.10. Statistical Analysis

All the data are presented as the mean + standard deviation (SD). All statistical analyses were performed using Easy R (EZR) software, version 1.40 [56]. The values below the detection limit in experiments are expressed as not detected. Statistical analysis of all data was performed by one-way analysis of variance, followed by post hoc comparisons with Tukey’s HSD test. All *p*-values < 0.05 between the groups were considered statistically significant.

## Data Availability

All data presented in this paper are available in the manuscript or from the corresponding author (H.Y.).

## Supplementary Materials

The following supporting information can be downloaded at https://www.mdpi.com/article/10.3390/ijms252011089/s1.

## References

[1] Kamiya H., Himeno T., Watarai A., Baba M., Nishimura R., Tajima N., Nakamura J. (2024). Prevalence and characteristics of diabetic symmetric sensorimotor polyneuropathy in Japanese patients with type 2 diabetes: The Japan Diabetes Complication and its Prevention Prospective study (JDCP study 10). J. Diabetes Investig..

[2] Akamine T., Takaku S., Suzuki M., Niimi N., Yako H., Matoba K., Kawanami D., Utsunomiya K., Nishimura R., Sango K. (2020). Glycolaldehyde induces sensory neuron death through activation of the c-Jun N-terminal kinase and p-38 MAP kinase pathways. Histochem. Cell Biol..

[3] Niimi N., Yako H., Takaku S., Kato H., Matsumoto T., Nishito Y., Watabe K., Ogasawara S., Mizukami H., Yagihashi S. (2018). A spontaneously immortalized Schwann cell line from aldose reductase-deficient mice as a useful tool for studying polyol pathway and aldehyde metabolism. J. Neurochem..

[4] Kato A., Tatsumi Y., Yako H., Sango K., Himeno T., Kondo M., Kato Y., Kamiya H., Nakamura J., Kato K. (2019). Recurrent short-term hypoglycemia and hyperglycemia induce apoptosis and oxidative stress via the ER stress response in immortalized adult mouse Schwann (IMS32) cells. Neurosci. Res..

[5] Yako H., Niimi N., Takaku S., Sango K. (2023). Advantages of omics approaches for elucidating metabolic changes in diabetic peripheral neuropathy. Front. Endocrinol..

[6] Nihei W., Kato A., Himeno T., Kondo M., Nakamura J., Kamiya H., Sango K., Kato K. (2024). Hyperglycaemia Aggravates Oxidised Low-Density Lipoprotein-Induced Schwann Cell Death via Hyperactivation of Toll-like Receptor 4. Neurol. Int..

[7] Li F., Szabo C., Pacher P., Southan G.J., Abatan O.I., Charniauskaya T., Stevens M.J., Obrosova I.G. (2004). Evaluation of orally active poly(ADP-ribose) polymerase inhibitor in streptozotocin-diabetic rat model of early peripheral neuropathy. Diabetologia.

[8] Obrosova I.G., Li F., Omorodola I.A., Forsell M.A., Komjáti K., Pacher P., Szabó C., Stevens M.J. (2004). Role of Poly(ADP-Ribose) Polymerase Activation in Diabetic Neuropathy. Diabetes.

[9] Obrosova I.G., Drel V.R., Pacher P., Ilnytska O., Wang Z.Q., Stevens M.J., Yorek M.A. (2005). Oxidative-nitrosative stress and poly(ADP-ribose) polymerase (PARP) activation in experimental diabetic neuropathy: The relation is revisited. Diabetes.

[10] Obrosova I.G., Pacher P., Szabo C., Zsengeller Z., Hirooka H., Stevens M.J., Yorek M.A. (2005). Aldose reductase inhibition counteracts oxidative-nitrosative stress and poly(ADP-ribose) polymerase activation in tissue sites for diabetes complications. Diabetes.

[11] Ho E.C., Lam K.S., Chen Y.S., Yip J.C., Arvindakshan M., Yamagishi S., Yagihashi S., Oates P.J., Ellery C.A., Chung S.S. (2006). Aldose reductase-deficient mice are protected from delayed motor nerve conduction velocity, increased c-Jun NH2-terminal kinase activation, depletion of reduced glutathione, increased superoxide accumulation, and DNA damage. Diabetes.

[12] Li F., Drel V.R., Szabo C., Stevens M.J., Obrosova I.G. (2005). Low-dose poly(ADP-ribose) polymerase inhibitor-containing combination therapies reverse early peripheral diabetic neuropathy. Diabetes.

[13] Negi G., Kumar A., Sharma S.S. (2010). Concurrent targeting of nitrosative stress-PARP pathway corrects functional, behavioral and biochemical deficits in experimental diabetic neuropathy. Biochem. Biophys. Res. Commun..

[14] Drel V.R., Lupachyk S., Shevalye H., Vareniuk I., Xu W., Zhang J., Delamere N.A., Shahidullah M., Slusher B., Obrosova I.G. (2010). New therapeutic and biomarker discovery for peripheral diabetic neuropathy: PARP inhibitor, nitrotyrosine, and tumor necrosis factor-alpha. Endocrinology.

[15] Zilberter Y., Gubkina O., Ivanov A.I. (2015). A unique array of neuroprotective effects of pyruvate in neuropathology. Front. Neurosci..

[16] Guarino V.A., Oldham W.M., Loscalzo J., Zhang Y.Y. (2019). Reaction rate of pyruvate and hydrogen peroxide: Assessing antioxidant capacity of pyruvate under biological conditions. Sci. Rep..

[17] Hegde K.R., Varma S.D. (2005). Prevention of cataract by pyruvate in experimentally diabetic mice. Mol. Cell. Biochem..

[18] Hegde K., Kovtun S., Varma S. (2011). Prevention of cataract in diabetic mice by topical pyruvate. Clin. Ophthalmol..

[19] Ju K.D., Shin E.K., Cho E.J., Yoon H.B., Kim H.S., Kim H., Yang J., Hwang Y.H., Ahn C., Oh K.H. (2012). Ethyl pyruvate ameliorates albuminuria and glomerular injury in the animal model of diabetic nephropathy. Am. J. Physiol. Ren. Physiol..

[20] Zhang X.M., Deng H., Tong J.D., Wang Y.Z., Ning X.C., Yang X.H., Zhou F.Q., Jin H.M. (2020). Pyruvate-Enriched Oral Rehydration Solution Improves Glucometabolic Disorders in the Kidneys of Diabetic db/db Mice. J. Diabetes Res..

[21] Yako H., Niimi N., Kato A., Takaku S., Tatsumi Y., Nishito Y., Kato K., Sango K. (2021). Role of pyruvate in maintaining cell viability and energy production under high-glucose conditions. Sci. Rep..

[22] Lopez-Fabuel I., Le Douce J., Logan A., James A.M., Bonvento G., Murphy M.P., Almeida A., Bolanos J.P. (2016). Complex I assembly into supercomplexes determines differential mitochondrial ROS production in neurons and astrocytes. Proc. Natl. Acad. Sci. USA.

[23] Maranzana E., Barbero G., Falasca A.I., Lenaz G., Genova M.L. (2013). Mitochondrial respiratory supercomplex association limits production of reactive oxygen species from complex I. Antioxid. Redox Signal..

[24] Bianchi C., Genova M.L., Parenti Castelli G., Lenaz G. (2004). The mitochondrial respiratory chain is partially organized in a supercomplex assembly: Kinetic evidence using flux control analysis. J. Biol. Chem..

[25] Lapuente-Brun E., Moreno-Loshuertos R., Acin-Perez R., Latorre-Pellicer A., Colas C., Balsa E., Perales-Clemente E., Quiros P.M., Calvo E., Rodriguez-Hernandez M.A. (2013). Supercomplex assembly determines electron flux in the mitochondrial electron transport chain. Science.

[26] Jha P., Wang X., Auwerx J. (2016). Analysis of Mitochondrial Respiratory Chain Supercomplexes Using Blue Native Polyacrylamide Gel Electrophoresis (BN-PAGE). Curr. Protoc. Mouse Biol..

[27] Park S., Jeon J.H., Min B.K., Ha C.M., Thoudam T., Park B.Y., Lee I.K. (2018). Role of the Pyruvate Dehydrogenase Complex in Metabolic Remodeling: Differential Pyruvate Dehydrogenase Complex Functions in Metabolism. Diabetes Metab. J..

[28] Rahman M.H., Jha M.K., Kim J.H., Nam Y., Lee M.G., Go Y., Harris R.A., Park D.H., Kook H., Lee I.K. (2016). Pyruvate Dehydrogenase Kinase-mediated Glycolytic Metabolic Shift in the Dorsal Root Ganglion Drives Painful Diabetic Neuropathy. J. Biol. Chem..

[29] Du X., Matsumura T., Edelstein D., Rossetti L., Zsengeller Z., Szabo C., Brownlee M. (2003). Inhibition of GAPDH activity by poly(ADP-ribose) polymerase activates three major pathways of hyperglycemic damage in endothelial cells. J. Clin. Investig..

[30] Vyas S., Chesarone-Cataldo M., Todorova T., Huang Y.H., Chang P. (2013). A systematic analysis of the PARP protein family identifies new functions critical for cell physiology. Nat. Commun..

[31] Weaver A.N., Yang E.S. (2013). Beyond DNA Repair: Additional Functions of PARP-1 in Cancer. Front. Oncol..

[32] Wang Y., Kim N.S., Haince J.F., Kang H.C., David K.K., Andrabi S.A., Poirier G.G., Dawson V.L., Dawson T.M. (2011). Poly(ADP-ribose) (PAR) binding to apoptosis-inducing factor is critical for PAR polymerase-1-dependent cell death (parthanatos). Sci. Signal..

[33] Nakajima H., Kubo T., Ihara H., Hikida T., Danjo T., Nakatsuji M., Shahani N., Itakura M., Ono Y., Azuma Y.T. (2015). Nuclear-translocated Glyceraldehyde-3-phosphate Dehydrogenase Promotes Poly(ADP-ribose) Polymerase-1 Activation during Oxidative/Nitrosative Stress in Stroke. J. Biol. Chem..

[34] Wahlberg E., Karlberg T., Kouznetsova E., Markova N., Macchiarulo A., Thorsell A.G., Pol E., Frostell A., Ekblad T., Oncu D. (2012). Family-wide chemical profiling and structural analysis of PARP and tankyrase inhibitors. Nat. Biotechnol..

[35] Mayo E., Fabrizio G., Scarpa E., Stilla A., Dani N., Chiacchiera F., Kleine H., Attanasio F., Lüscher B., Di Girolamo M. (2018). ARTD10/PARP10 Induces ADP-Ribosylation of GAPDH and Recruits GAPDH into Cytosolic Membrane-Free Cell Bodies When Overexpressed in Mammalian Cells. Challenges.

[36] Leist M., Single B., Naumann H., Fava E., Simon B., Kuhnle S., Nicotera P. (1999). Inhibition of mitochondrial ATP generation by nitric oxide switches apoptosis to necrosis. Exp. Cell Res..

[37] Lieberthal W., Menza S.A., Levine J.S. (1998). Graded ATP depletion can cause necrosis or apoptosis of cultured mouse proximal tubular cells. Am. J. Physiol..

[38] Andrabi S.A., Umanah G.K., Chang C., Stevens D.A., Karuppagounder S.S., Gagne J.P., Poirier G.G., Dawson V.L., Dawson T.M. (2014). Poly(ADP-ribose) polymerase-dependent energy depletion occurs through inhibition of glycolysis. Proc. Natl. Acad. Sci. USA.

[39] Zamaraeva M.V., Sabirov R.Z., Maeno E., Ando-Akatsuka Y., Bessonova S.V., Okada Y. (2005). Cells die with increased cytosolic ATP during apoptosis: A bioluminescence study with intracellular luciferase. Cell Death Differ..

[40] Li T., Zhang Z., Kolwicz S.C., Abell L., Roe N.D., Kim M., Zhou B., Cao Y., Ritterhoff J., Gu H. (2017). Defective Branched-Chain Amino Acid Catabolism Disrupts Glucose Metabolism and Sensitizes the Heart to Ischemia-Reperfusion Injury. Cell Metab..

[41] Mizukami H., Osonoi S., Takaku S., Yamagishi S., Ogasawara S., Sango K., Chung S., Yagihashi S. (2020). Role of glucosamine in development of diabetic neuropathy independent of the aldose reductase pathway. Brain Commun..

[42] Hammes H.P., Du X., Edelstein D., Taguchi T., Matsumura T., Ju Q., Lin J., Bierhaus A., Nawroth P., Hannak D. (2003). Benfotiamine blocks three major pathways of hyperglycemic damage and prevents experimental diabetic retinopathy. Nat. Med..

[43] Park J., Chen Y., Tishkoff D.X., Peng C., Tan M., Dai L., Xie Z., Zhang Y., Zwaans B.M., Skinner M.E. (2013). SIRT5-mediated lysine desuccinylation impacts diverse metabolic pathways. Mol. Cell.

[44] Antoun G., McMurray F., Thrush A.B., Patten D.A., Peixoto A.C., Slack R.S., McPherson R., Dent R., Harper M.E. (2015). Impaired mitochondrial oxidative phosphorylation and supercomplex assembly in rectus abdominis muscle of diabetic obese individuals. Diabetologia.

[45] Alejandra Sanchez-Munoz M., Valdez-Solana M.A., Campos-Almazan M.I., Flores-Herrera O., Esparza-Perusquia M., Olvera-Sanchez S., Garcia-Arenas G., Avitia-Dominguez C., Tellez-Valencia A., Sierra-Campos E. (2018). Streptozotocin-Induced Adaptive Modification of Mitochondrial Supercomplexes in Liver of Wistar Rats and the Protective Effect of *Moringa oleifera* Lam. Biochem. Res. Int..

[46] Jeoung N.H., Harris C.R., Harris R.A. (2014). Regulation of pyruvate metabolism in metabolic-related diseases. Rev. Endocr. Metab. Disord..

[47] Hinder L.M., Vivekanandan-Giri A., McLean L.L., Pennathur S., Feldman E.L. (2013). Decreased glycolytic and tricarboxylic acid cycle intermediates coincide with peripheral nervous system oxidative stress in a murine model of type 2 diabetes. J. Endocrinol..

[48] Konrad T., Vicini P., Kusterer K., Hoflich A., Assadkhani A., Bohles H.J., Sewell A., Tritschler H.J., Cobelli C., Usadel K.H. (1999). alpha-Lipoic acid treatment decreases serum lactate and pyruvate concentrations and improves glucose effectiveness in lean and obese patients with type 2 diabetes. Diabetes Care.

[49] Lin H.T., Cheng M.L., Lo C.J., Lin G., Lin S.F., Yeh J.T., Ho H.Y., Lin J.R., Liu F.C. (2019). 1H Nuclear Magnetic Resonance (NMR)-Based Cerebrospinal Fluid and Plasma Metabolomic Analysis in Type 2 Diabetic Patients and Risk Prediction for Diabetic Microangiopathy. J. Clin. Med..

[50] Jin Q., Ma R.C.W. (2021). Metabolomics in Diabetes and Diabetic Complications: Insights from Epidemiological Studies. Cells.

[51] Gao H., Jiang Q., Ji H., Ning J., Li C., Zheng H. (2019). Type 1 diabetes induces cognitive dysfunction in rats associated with alterations of the gut microbiome and metabolomes in serum and hippocampus. Biochim. Biophys. Acta Mol. Basis Dis..

[52] Diao C., Zhao L., Guan M., Zheng Y., Chen M., Yang Y., Lin L., Chen W., Gao H. (2014). Systemic and characteristic metabolites in the serum of streptozotocin-induced diabetic rats at different stages as revealed by a 1H-NMR based metabonomic approach. Mol. Biosyst..

[53] Das B.K., Jayalakshmi K., Gadad P.C. (2022). 1H-NMR-based serum metabolomic study to evaluate the effect of asarone and metformin on experimentally induced diabetic hepatocellular carcinoma in rats. Bull. Natl. Res. Cent..

[54] Zhao L., Liu X., Xie L., Gao H., Lin D. (2010). 1H NMR-based metabonomic analysis of metabolic changes in streptozotocin-induced diabetic rats. Anal. Sci..

[55] Rawat A., Misra G., Saxena M., Tripathi S., Dubey D., Saxena S., Aggarwal A., Gupta V., Khan M.Y., Prakash A. (2018). 1H NMR Based Serum Metabolic Profiling Reveals Differentiating Biomarkers in Patients with Diabetes and Diabetes Comorbidity. Diabetes Metab. Syndr. Clin. Res. Rev..

[56] Kanda Y. (2013). Investigation of the freely available easy-to-use software ‘EZR’ for medical statistics. Bone Marrow Transplant..

