# Supplementary material for: Role of Exogenous Pyruvate in Maintaining Adenosine Triphosphate Production under High-Glucose Conditions through PARP-Dependent Glycolysis and PARP-Independent Tricarboxylic Acid Cycle
Source: Int J Mol Sci. 2024 Oct 15;25(20):11089. doi: 10.3390/ijms252011089 (PMC11508270; doi:10.3390/ijms252011089)
Supplement: Supplementary file 1 [file ijms-25-11089-s001.zip › ijms-3204232-supplementary.pdf]

Role of Exogenous Pyruvate in Maintaining Adenosine Triphosphate Production under High-Glucose Conditions through PARP-Dependent Glycolysis and PARP-Independent Tricarboxylic Acid Cycle

Hideji Yako, Naoko Niimi, Shizuka Takaku, Ayako Kato, Koichi Kato and Kazunori Sango

Supplementary Materials: Figure (6)

Figure S1

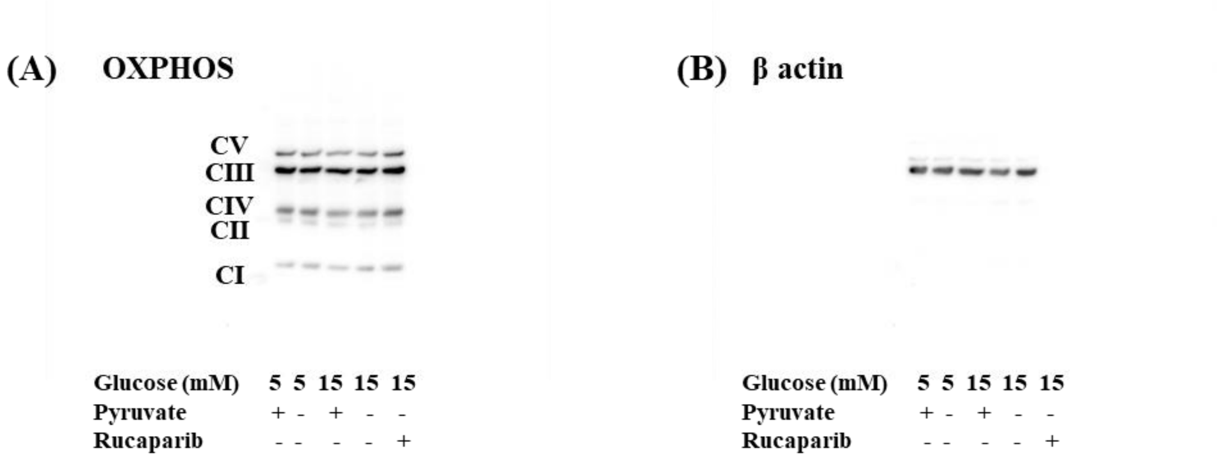

Figure S1. The actual Western blotting representative images of complex (C) I, II, III, IV and V, and β actin.
Full length of the membranes of CI (NDUFB8), CII (SDHB), CIII (MTCO1), CIV (UQCR2) and CV (ATP5A) (**A**) and β actin (**B**) presented in **Fig. 2B** were sequentially visualized in the same membranes. OXPHOS; oxidative phosphorylation.

Figure S2

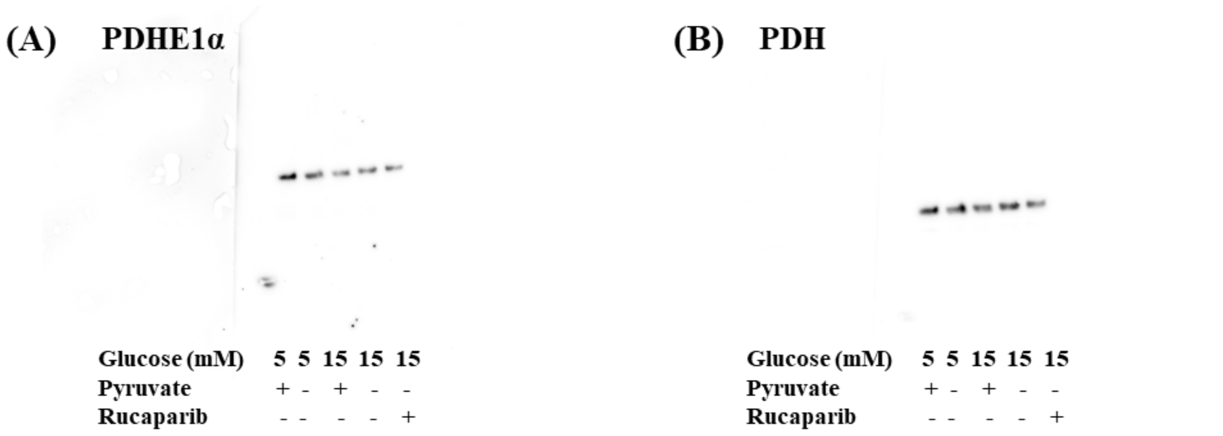

Figure S2. The actual Western blotting representative images of Phospho PDHE1α and PDH.
The full length of the membranes of Phospho PDHE1α (**A**) and PDH (**B**) presented in **Figure 3B** were sequentially visualized in the same membranes.

Figure S3

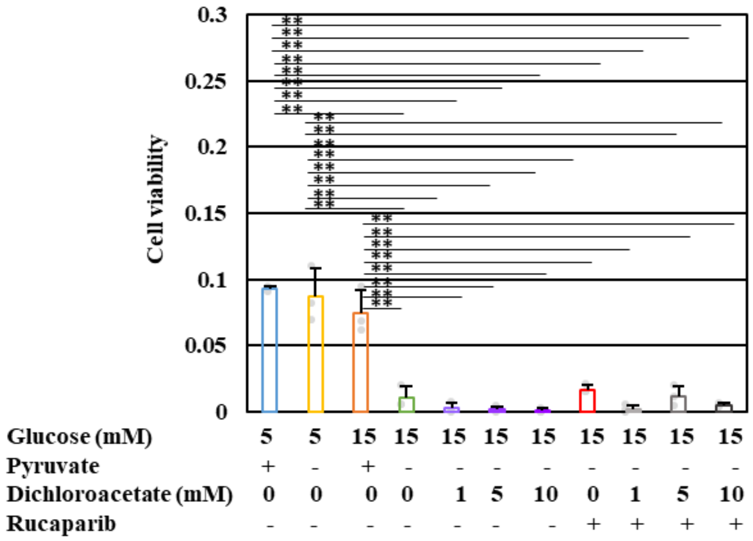

Figure S3. The effect of dichloroacetate (DCA) on the cell viability under high-glucose pyruvate-starved conditions.

IMS32 cell viability under 5 mM glucose in the presence (**blue**) and absence (**yellow**) of pyruvate conditions, 15 mM glucose in the presence (**brown**) and absence (**green**) of pyruvate conditions, and 15 mM glucose in the absence of pyruvate conditions supplemented with rucaparib (**red**), 1, 5, and 10 mM dichloroacetate (**purple**), and both rucaparib and dichloroacetate (**grey**) were assessed using MTS assay. The values represent mean + SD from three experiments (individual values are depicted as circles). ***P* < 0.01.

Figure S4

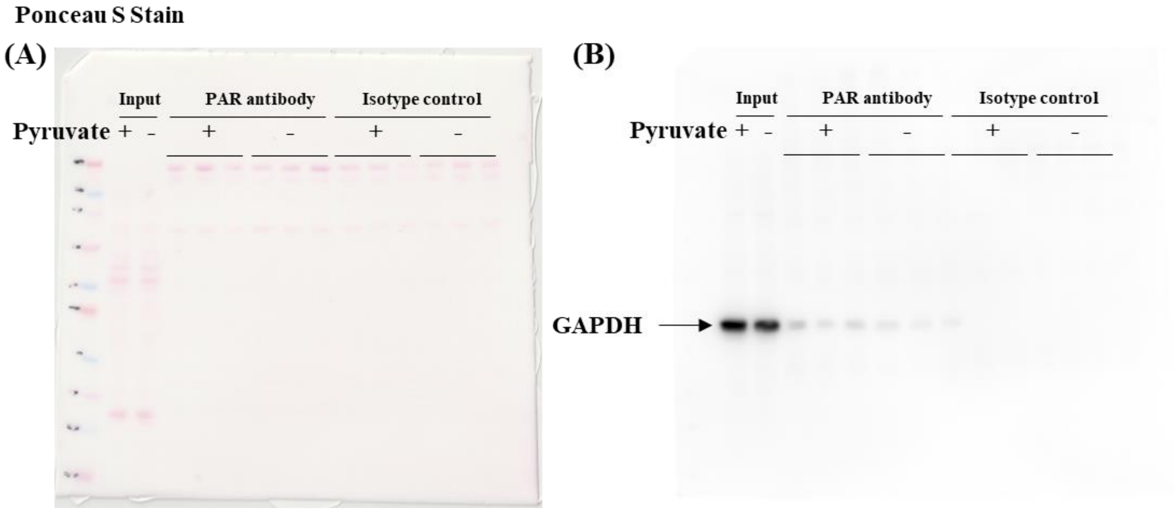

Figure S4. The actual ponceau S stain and Western blotting images of polyribosylated GAPDH.

The full length of the membranes of ponceau S stain (**A**) and GAPDH (**B**) presented in **Figure 5A** were sequentially visualized in the same membrane. PAR; poly (ADP-ribose) polymer.

Figure S5

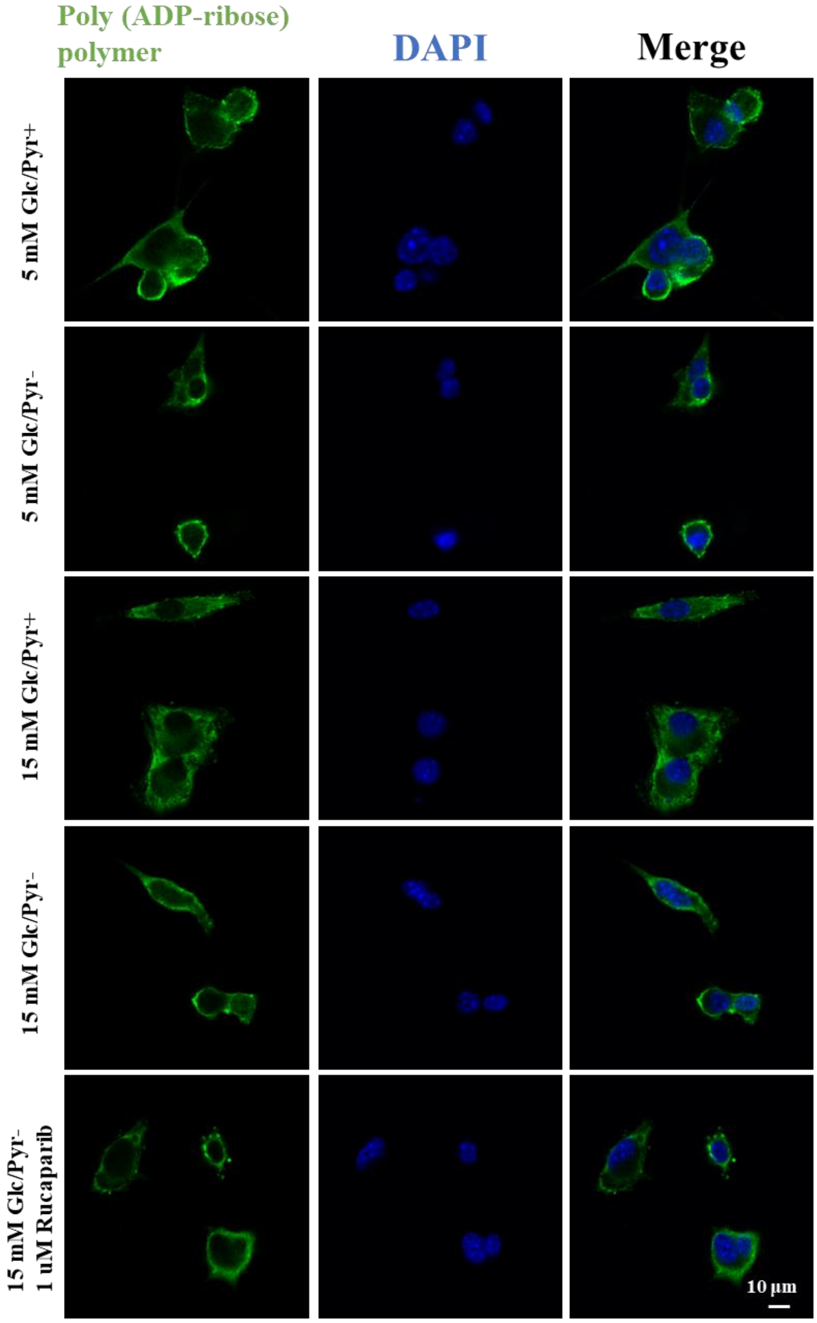

Figure S5. Immunocytochemistry of poly (ADP-ribose) polymer in IMS32 cells under normal- and high-glucose conditions in the presence and absence of pyruvate supplemented with rucaparib. Images of poly (ADP-ribose) polymer (**green**), nuclei (**DAPI; blue**) and merge in IMS32 cells exposed to 5 mM glucose in the presence and absence of pyruvate conditions, 15 mM glucose in the presence and absence of pyruvate conditions, and 15 mM glucose in the absence of pyruvate conditions supplemented with rucaparib. Scale bar: 10 μm.

Figure S6

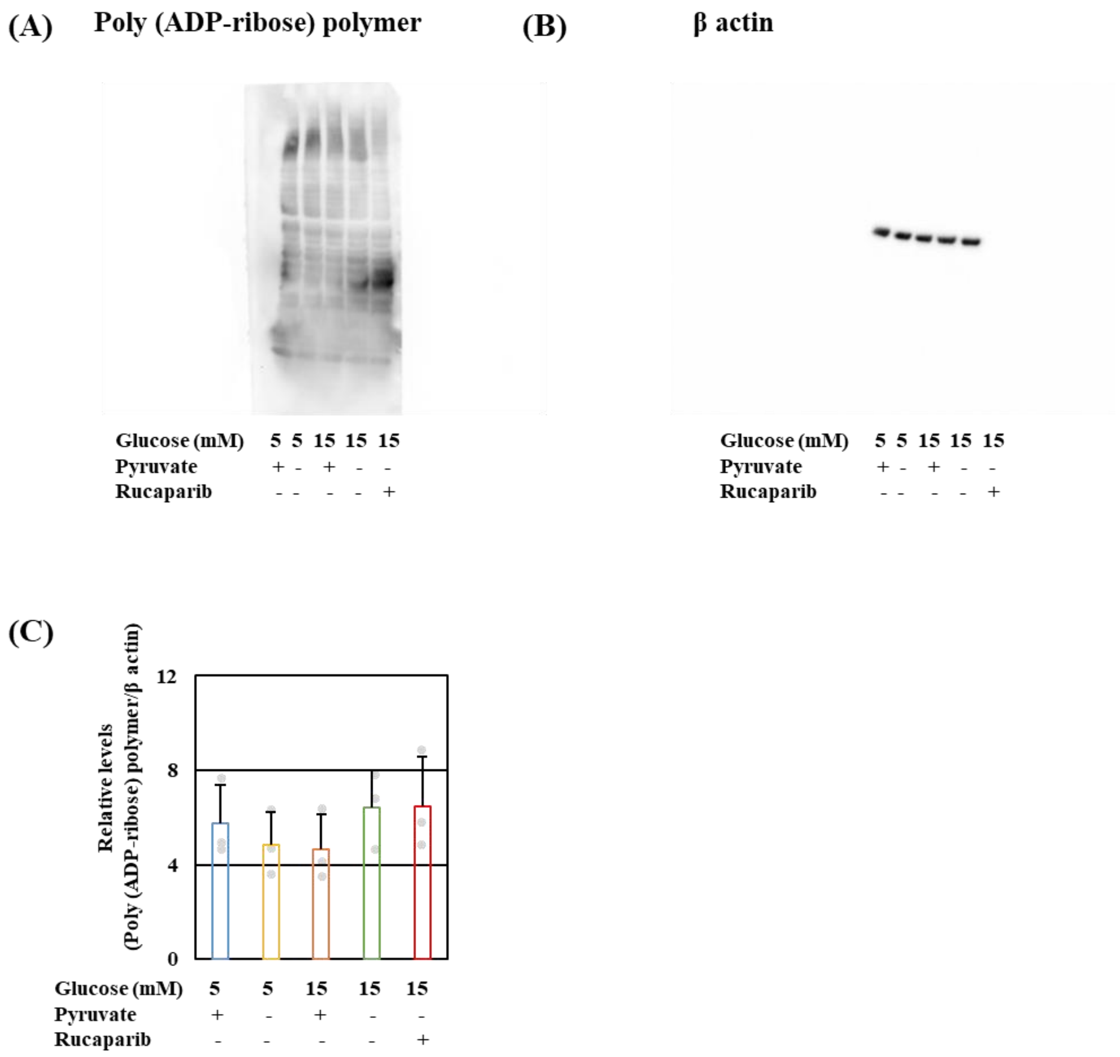

Figure S6. The levels of poly (ADP-ribose) polymer were not changed under high-glucose pyruvate-starved conditions.

The full length of the membranes of poly (ADP-ribose) polymer (**A**) and β actin (**B**) were sequentially visualized in the same membrane. Poly (ADP-ribose) polymer (**C**) of IMS32 cell under exposure to 5 mM glucose in the presence (**blue**) and absence (**yellow**) of pyruvate conditions, and 15 mM glucose in the presence (**brown**) and absence (**green**) of pyruvate conditions containing rucaparib (**red**) were determined. The values represent mean + SD from three experiments (individual values are depicted as circles).
